# Entries, Winter Session, 1890-91

**Published:** 1890-11-01

**Authors:** 


### THE MEDICAL SCHOOLS.

### ENTRIES, WINTER SESSION, 1890-91.

We give below a list of the number of students who have joined the various schools of medicine this session. Of the London hospitals, St. Bartholomew's has the greatest, Guy's coming next in order. Some of the provincial schools are not yet complete, but the entries at Cambridge so far are only exceeded by one London hospital, namely, Barts, and it must be remembered that these university numbers by no means represent the whole of the students who have just entered with the idea of joining the profession. Many men prefer to enter and take their arts degree in other subjects, classics or mathematics, first, and then to commence their medical studies.

| | A. | B. | C. | D. | E. |
|---|---|---|---|---|---|
| St. Bartholomew's Hospital | 127 | 27 | 0 | * | 154 |
| Charing Cross Hospital | 31 | 14 | 35 | — | 80 |
| St. George's Hospital | 30 | 3 | 0 | — | 33 |
| Guy's Hospital | 101 | 31 | 16 | 16 | 148 |
| King's College | 30 | 57 | 0 | 11 | 87 |
| London Hospital | 59 | 30 | — | 9 | 89 |
| St. Mary's Hospital | 33 | 16 | — | 5 | 47 |
| Middlesex Hospital | 50 | 59 | 8 | 6 | 115 |
| St. Thomas's Hospital | 66 | 34 | — | 7 | 100 |
| University College | 67 | 21 | 0 | 65 | 88 |
| Westminster Hospital | 21 | 1 | 1 | — | 23 |
| London School of Medicine for Women | 34 | 4 | — | — | 38 |
| Cambridge University | 117 | — | — | — | 110 |
| Oxford University§ | | | | | |
| University of Durham College of Medicine | 34 | 126 | 1 | — | 161 |
| Bristol Medical School | 17 | 2 | — | * | 19 |
| Owen's College, Manchester | 86 | 32 | 6 | 37 | 124 |
| University College, Liverpool | 26 | — | — | 6 | 26 |
| Yorkshire College, Leeds | 35 | 4 | 0 | 15 | 39 |
| Queen's College, Birmingham | 41 | 32 | 7 | 4 | 80 |
| London School of Dental Surgery | — | — | 34 | — | — |

A. Number of students who have entered for full curriculum.
B. Number of students who have joined for special courses.
C. Number of dental students.
D. Number of students who have joined classes for preliminary scientific instruction.
E. Total, excluding students attending classes for preliminary scientific instruction.
* In compliance with the regulations of the University of London, students attending these classes are not counted as medical students.
§ Numbers not at present obtainable.

